# Functional Genomics at the *Arabidopsis* Meeting

**DOI:** 10.1002/1097-0061(20000930)17:3<235::AID-YEA36>3.0.CO;2-X

**Published:** 2000

**Authors:** Karin van de Sande, Ottoline Leyser

**Affiliations:** Department of Biology University of York Heslington York YO10 5DD UK


**Yeast**
*Yeast* 2000; **17**: 235–237.

**Meeting Review**

### Functional genomics at the *Arabidopsis* meeting

### The 11th International Conference on *Arabidopsis* Research, University of Wisconsin, Madison, USA. June 2000

Karin van de Sande* and Ottoline Leyser
*Department of Biology, University of York, Heslington, York YO10 5DD, UK*

**Correspondence to:*
*Karin van de Sande,*
*Department of Biology,*
*University of York, Heslington,*
*York YO10 5DD, UK.*
*E-mail: kvd1@york.ac.uk*

**Keywords: Arabidopsis; meeting overview; functional genomics; microarray; expression profiling**

The 11th International Conference on *Arabidopsis* Research (June 24–28) took place at the University of Wisconsin in Madison. A wide range of topics was covered in 18 sessions, ranging from 'genomics' to 'floral' induction, and 'patterning and organogenesis'. In the evenings, several very popular genomics workshops took place. Chuck Gasser (University of California) led the workshop, 'Discussion of the 2010 Project', and workshops and drop-in sessions were organized by TAIR (The *Arabidopsis* Information Resource) and AFGC (*Arabidopsis* Functional Genomics Consortium). Invariably, even after rescheduling to a larger room, the rooms were crowded with many people standing or sitting on the floor.

Starting with the Keynote Address, Richard Jefferson spoke on the global impact of plant biology. He stimulated people to 'harness the creative force in *Arabidopsis* biology for the public good' and passionately pleaded for a reduction of restrictions to the application of biotechnology in agriculture. Cambia, Jefferson's Australia-based biotech company, is developing new methods to use biotechnology in plant breeding. He has started a transgenomics initiative using expression profiling for genetic diversity analysis of uncharacterized germplasm and fast building of genetic maps. Using transcriptional activator facilitated enhancer trapping (TAFET), targeted evolution of novel plant traits is addressed. More than 4000 TAFET rice lines have been developed thus far.

An overview of the progress on various genome grants was given in the first session, chaired by Jeff Dangl (University of North Carolina). Short reports on NSF (National Science Foundation) grants gave an update on different projects providing tools for the *Arabidopsis* community.

Judith Bender (Johns Hopkins University) described a project on the functional genomics of chromatin, which is now mid-way through the first year. Database mining and reverse genetics, with emphasis on gene silencing, will be used for maize and *Arabidopsis*. At **http://ag.arizona.edu/chromatin/chromatin.html** information can be found on targeted chromatin genes, the dsRNA vector system used and on chromatin mutant strains.

Andrew Brent (University of Wisconsin) presented the use of functional and comparative genomics in plant pathogen interactions. Roughly 1% of *Arabidopsis* genes encode NBS–LRR (nucleotide binding site–leucine-rich repeat) proteins, a combination that to date has only been found in plant disease resistance genes (R genes). To resolve the complex signal transduction involved, expression profiling is being used, using the Affymetrix chip. In this way it is checked, for instance, what genes are induced by the action of different R gene products or by pathogens or their proteins. This

Copyright © 2000 John Wiley & Sons, Ltd.

approach will be complemented by overexpression studies.

Ray Bressan (Purdue University) talked about the Stress Functional Genomics Consortium (University of Arizona, Oklahoma State University, Purdue University). 25 000 ESTs of salt-induced genes are generated in *Arabidopsis*, 40% of which were not yet in the database. An 18 000 spot microarray is being made using 3000 ESTs from sodium chloride-treated material and 15 000 ESTs made available by Tom Newman (MSU–DOE Plant Research Laboratory, Michigan State University). T-DNA mutants are being made using insertion vectors and activation tagging vectors, and seeds will be pooled for reverse genetics. These mutants are being screened for different stress responses and thus far, among others, two salt-sensitive and five salt-resistant mutants have been found. Ultimately all the lines will go to the stock centre, together with DNA pools.

Pam Green (Michigan State University) reported on AFGC, which has two services, microarray and T-DNA knockout. The project consists of technology development, but also carries out research. The knockout service opened in October 1999 for US users and in January 2000 for international users. The microarray service produces slides with 11 000 clones, of which an estimated 8500 are unique ESTs. The second cycle of proposals has just been accepted and data from the first experiments are available at the AFCG website.

John Quackenbush presented TIGR's whole chromosome gene expression analysis in *Arabidopsis*, using DNA microarray. TIGR finished sequencing chromosome 2 and is now working on validation of gene predictions. The 3′ ends of the predicted genes of chromosome 2 were amplified with a very impressive success rate and used for the microarray. In parallel a clone-based array strategy is carried out. Information on optimalization of the microarray methods used will soon be available on the TIGR website and the software that was developed is already available. From chromosome 2 only 25% of the genes were represented in the ESTs thus far.

Sakis Theologis (Plant Gene Expression Center, PGEC) represented the SSP consortium (Stanford University, Salk Institute and PGEC) that is working together with Affymetrix on a chip containing all full-length *Arabidopsis* genes. This will allow the finding of new, expressed genes that are not among the ESTs, the experimental verification of annotation, and the use of the chip in expression studies.

John Walker (University of Missouri) discussed functional genomics of kinases, with extensive bioinformatics and insertion mutagenesis; information available on **http://plantsp.sdsc.edu**.

Nan Eckardt (Pennsylvania State University) discussed the use of PCR suppression subtractive hybridization libraries in the collection of stress cDNAs in different plant defence responses. Specialized cDNA microarrays, the stress chips, were prepared from these clones and from ESTs from several known stress-induced genes, and hybridized with RNA from plants harvested at different times after various pathogen or stress treatments. It was found that 'simple and similar patterns are observed in widely disparate systems'. Therefore, complex gene systems exhibit a simple continuous response to perturbation. There were a few posters from the same project, for instance one from Mali Mahalingam from Nina Fedoroff's lab, working on biotropic fungal pathogens such as *Peronospora parasitica*. **http://sg102.biotec.psu.edu** gives more information on the project.

In several other presentations and posters, the use of microarrays was discussed. Steve Whitham (NADI) used the Affymetrix gene chip for the identification of genes induced by virus infection, finding several genes involved in biotic and abiotic stress responses, encoding metabolic proteins or heat shock proteins, kinases and receptor kinases and several with unknown function. Their project is moving on to targeting several of the found genes with reverse genetics, to determine their role in infection.

Doug Boyes (Paradigm Genetics) introduced a high-throughput functional genomics project combining detailed phenotype analysis with metabolite profiling and microarray analysis. Ultimately, growth stage-specific links will be made between these data.

The multinational coordinated *Arabidopsis* project started as a follow-up to the *Arabidopsis* genome sequencing efforts. It wants to give direction to future *Arabidopsis* research and to stimulate and make full use of new developments in plant genomics. The workshop on the multinational coordinated *Arabidopsis* 2010 project was a follow-up of two workshops on this subject that took place earlier this year, for which reports can be found at the TAIR website (**http://www.arabidopsis.org**).

Copyright © 2000 John Wiley & Sons, Ltd. 

Detlef Weigel presented an overview of the 10-year goals to be reached, and asked for input from the community. With a final aim of knowing every molecule at any time in every cell, a virtual plant can be created from which can be predicted what happens when specific changes are introduced. The 2010 project, which is even more ambitious than the genome-sequencing project, is divided into short- (1–3 years), mid- (3–6 years) and long-term (6–10 years) aims. Some of the goals for the short term are knockout mutants and full-length cDNAs of all genes and subcellular localization for all proteins. For the mid-term, global metabolite pictures, expression patterns for all genes and development of methods for directed genetics are envisaged. Some long-term goals are construction of plant artificial chromosomes, knowing cis regulatory sequences for all genes and biochemical functions for all proteins. A large proportion of these resources will be generated in centres that develop the technology and make it available for everybody.

Different European initiatives then were presented, showing the worldwide importance of functional genomics. Thomas Altmann (MPI-Golm) presented Genome Analysis in Biological System Plant (GABI). Initially funded for 3–4 years, with research in *Arabidopsis*, barley, rapeseed, maize, rye, poplar and sugar beet, GABI is funded partly governmentally, partly industrially. Apart from several resource centres, many research groups are part of the project. Proteomics, microarrays, protein arrays, yeast two-hybrid, an *Arabidopsis* knockout facility and bioinformatics will form part of the resources. Kiyotaka Okada introduced the Japanese functional genomics projects. The Kazusa DNA research Institute, Riken Genomic Science Centre and the Riken Plant Research Centre will be involved in microarrays, production of tDNA and transposon-tagged lines and collection and construction of mutants. Liz Dennis represented the Australian approach, explaining that several individual groups are involved in small projects, but that funding from the government for national projects was lacking. CSIRO is involved in microarrays and development of new systems for gene silencing. Pierre Hilson introduced Genoplante. A 5 year French programme, it consists of different companies and public institutions. Genoplante will work on *Arabidopsis* and different crop plants, for instance developing functional genomics on the model genomes rice and *Arabidopsis*, and analysing synteny between major crops and model genomes. Pierre stressed the importance of international standards for data collection and access. Sean May introduced the UK functional genomics project, called GARNet. GARNet, the Genomic *Arabidopsis* Resource Network, will be funded for 3 years by the BBSRC (Biology and Biotechnological Sciences Research Council) and is aimed purely at creating services and resources. The GARNet project consists of the sequencing of transposon insertion sites, creating mutant lines with multiple transposon insertions, screening of large insert cosmid libraries, proteomics, metabolomics and transcriptomics (macro- and microarray). Information on GARNet, and a meeting organized for potential users of the different services, can be found at **http://garnet.Arabidopsis.org.uk**.

The crowded AFGC workshops first introduced the AFGC microarray services, explaining the procedures followed when one applies for service, quality controls and how to use the database of microarray results that is now available. In the second workshop, several people presented data obtained using microarrays, including the Stress microarray chip as used by Nina Fedorofs' lab, which involves clones from Monsanto, AFGC's own 'biological conditions' experiments with data on their circadian rhythm experiments, the NADI programme involving Novartis, and the Affymetrix gene chip, which is available now. These presentations show that the use of tools of functional genomics in *Arabidopsis* is increasing at a rapid pace. Undoubtedly, next year even more very interesting results will be available.

Copyright © 2000 John Wiley & Sons, Ltd. 

